# Capsaicin suppresses hepatocarcinogenesis by inhibiting the stemness of hepatic progenitor cells via SIRT1/SOX2 signaling pathway

**DOI:** 10.1002/cam4.4777

**Published:** 2022-06-08

**Authors:** Zhi‐Qin Xie, Hong‐Xia Li, Xiao‐Juan Hou, Mei‐Yuan Huang, Ze‐Min Zhu, Li‐Xin Wei, Cai‐Xi Tang

**Affiliations:** ^1^ Department of Hepatobiliary and Pancreatic Surgery Zhuzhou Hospital Affiliated to Xiangya School of Medicine, Central South University Zhuzhou City Hunan Province China; ^2^ Department of Pathology, Zhuzhou Hospital Affiliated to Xiangya School of Medicine Central South University Zhuzhou City Hunan Province China; ^3^ Tumor Immunology and Gene Therapy Center, Eastern Hepatobiliary Surgery Hospital The Second Military Medical University Shanghai City China

**Keywords:** capsaicin, hepatic progenitor cells, hepatocellular carcinoma, SIRT1, SOX2

## Abstract

**Background & Aims:**

Capsaicin, a functional component of chili pepper, possesses anti‐inflammatory, analgesic, and anti‐cancer properties. This study aimed to determine the property of capsaicin against hepatocarcinogenesis in vivo and investigate the role of the SIRT1/SOX2 pathway in the mode of action of capsaicin in hepatic progenitor cells (HPCs), which is related to hepatocarcinogenesis.

**Materials & Methods:**

We prepared a diethylnitrosamine‐induced liver cancer model in rats to examine hepatocarcinogenesis, and delivered liposomal capsaicin through the subcutaneous transposition of the spleen to the liver. Liver sections from rats and hepatocarcinoma patients were stained for the markers of HPCs or SIRT1/SOX2 signaling. SIRT1/SOX2 signalling expression was measured using immunoprecipitation and western blot.

**Results:**

We found that capsaicin significantly inhibited hepatocarcinogenesis. Notably, capsaicin inhibited HPCs activation in vivo but did not induce apoptosis in the normal hepatic progenitor cell line in rats in vitro. This suggests that capsaicin suppresses hepatocarcinogenesis by inhibiting the stemness of HPCs. Moreover, capsaicin can induce this inhibition by reducing the stability of SOX2. SIRT1 is overexpressed in liver cancer and acts as a tumor promoter via SOX2 deacetylation. Using immunoprecipitation, we identified direct binding between SIRT1 and SOX2. The capsaicin treatment resulted in SIRT1 downregulation which reduced deacetylation, and increased nuclear export as well as subsequent ubiquitous degradation of SOX2.

**Conclusions:**

Altogether, we report that capsaicin suppresses hepatocarcinogenesis by inhibiting the stemness of HPCs via SIRT1/SOX2 signaling. It may serve as a promising therapeutic candidate for liver cancer.

## Graphical abstract

- Capsaicin inhibits stemness of hepatic progenitor cells via SIRT1/SOX2 signaling.
- Capsaicin suppresses hepatocarcinogenesis.
- Capsaicin treatment results in SIRT1 downregulation.
- Capsaicin may be a promising therapeutic candidate for liver cancer.

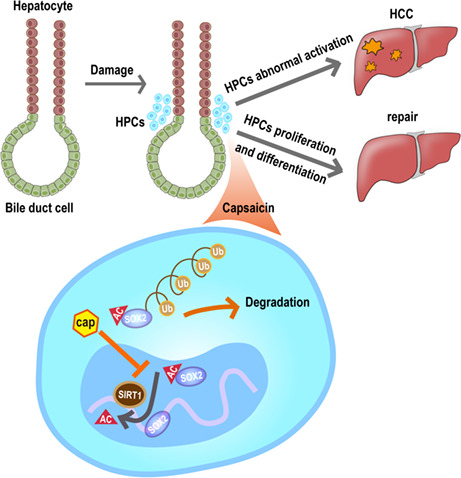

## 1 INTRODUCTION

Hepatocellular carcinoma (HCC) is the sixth most common cancer and the third leading cause of cancer mortality worldwide.^1^ Accumulating evidence indicates that hepatic progenitor cells (HPCs) are capable of liver regeneration and differentiation, and are pivotal sources of tumor initiation.^2^, ^3^ The balance between self‐renewal and differentiation of HPCs is essential for supplying the liver with specific hepatobiliary populations, both under physiological and pathological conditions.^4^ The early stage of HCC generation is often accompanied by HPCs activation under continuous injury and chronic inflammation. HPCs have bipotent differentiation capability and can transform into HCC or intrahepatic cholangiocarcinoma under abnormal activation conditions.^5^ Thus, disruption of HPCs activation might be a potential target for the treatment of HCC.

The use of natural products is among the many strategies for cancer prevention. Capsaicin, a functional component of chili pepper, possesses anti‐inflammatory, analgesic, and anti‐cancer properties and acts against liver, colon, and breast cancers.^6^, ^8^ Capsaicin is a highly selective agonist of the TRPV1 receptor, and therefore it has been widely used as a food additive and pharmacological intervention for neuropathic pain.^9^ A recent study showed that capsaicin displays antitumor effects in HCC cell lines and enhances the sensitivity of a chemotherapy drug.^10^ However, the anticancer potential of capsaicin in HCC has rarely been explored in animal models.

SOX2 is a transcriptional factor that maintains the stemness of embryonic stem cells, progenitor cells, and cancer stem cells (CSCs).^11^, ^12^ Although the role of SOX2 in regulating liver CSCs has been extensively studied, it remains unclear whether SOX2 is involved in the regulation of HPCs stability.^13^ Post‐translational modifications of SOX2 are important factors in regulating pluripotency. Phosphorylation of SOX2 at threonine 116 stabilizes SOX2, promoting esophageal cancer cell proliferation and stemness.^14^ Acetylation of SOX2 is required in mouse somatic reprogramming and multipotency in mesenchymal stem cells.^15^, ^16^ However, in HPCs, the mechanisms that stabilize SOX2 by post‐translational modifications remain unknown. SIRT1, an NAD‐dependent deacetylase, contributes to tumor progression by maintaining the self‐renewal of CSCs and promoting carcinogenesis.^13^ Overexpression of SIRT1 induces HCC tumorigenesis and strengthens resistance to chemotherapy.^17^, ^18^ This study demonstrated that SIRT1 was overexpressed in HCC and decreased after capsaicin treatment in vivo and in vitro. However, the role of SIRT1 in the regulation of HPCs and carcinogenesis has not yet been elucidated.

In this study, we investigated the role of SIRT1 in maintaining the stemness of HPCs, especially focusing on its ability to deacetylate SOX2. We found that following capsaicin treatment, SIRT1 is downregulated, and then acetylated SOX2 is translocated from the nucleus to the cytosol, and degraded by ubiquitination. This study showed that capsaicin inhibited early‐stage tumorigenesis in diethylnitrosamine (DEN)‐induced rat HCC by inhibiting HPCs activation.

## 2 MATERIALS AND METHODS

### 2.1 Preparation of liposomes

Preparation and detection of liposomes were performed as previously described.^19^ Capsaicin, L‐α‐phosphatidylcholine (LP), cholesterol (CHL), and dicetyl phosphate (DCP) were dissolved in chloroform in round bottom flasks (250 ml). The solution was evaporated at 60°C in a rotary evaporator at a rotation speed of 120 rpm for 90 min, and a thin film was formed. The thin film was hydrated with phosphate‐buffered saline (PBS) (pH = 7.4) at 60°C for 30 min in a rotary evaporator with a rotation speed of 120 rpm until the lipid film was completely dissolved. The mixture was sonicated for 15 min to reduce the vesicle size. Liposomal capsaicin with an average diameter of 140 nm, zeta potential of −24.97 mV, and encapsulation efficiency of 66% was prepared.

### 2.2 Cells and reagents

WB‐F344 cells and HepG2 cells were incubated in Dulbecco's modified Eagle's medium (DMEM), containing 10% fetal bovine serum (FBS, Gibco) and 100 mg/L penicillin (Gibco) at 37°C in a humidified atmosphere containing 5% CO_2_. MG‐132, LP, CHL, DCP, and capsaicin were purchased from Sigma‐Aldrich (Darmstadt, Germany). Tunnel apoptosis detection kits were purchased from MBL.

### 2.3 Animal experiments

Male Sprague–Dawley rats (6–7 weeks old, 160–180 g) were obtained from Shanghai Laboratory Animal Center (Shanghai, China), and were housed in a pathogen‐free animal facility. To induce HCC, rats received 0.01% DEN (Sigma‐Aldrich) through drinking water. At 8 weeks after DEN treatment, liposomal capsaicin was administered twice a week at 1 mg/kg in a low dose group and at 2 mg/kg in a high dose group for 4–6 weeks via subcutaneously transplanted spleen. At 12–14 weeks after DEN treatment, animals were sacrificed and tumors were obtained. All visible nodules on the liver surface were counted. Human specimens were obtained from hepatocarcinoma patients at the pathology department. Specimens were fixed in 10% formalin and embedded in paraffin for histopathological analysis, immunofluorescent staining, and immunohistochemistry assays. All experiments procedures were conducted according to the protocols approved by the Ethics Committee of Zhuzhou Hospital Affiliated to Xiangya School of Medicine and Animal Ethics Committee of the Second Xiangya Hospital, Central South University. Informed consent was obtained from all patients. All research was performed in accordance with the relevant guidelines and regulations.

### 2.4 Immunohistochemical analysis

Formalin‐fixed and paraffin‐embedded liver sections were blocked in 5% bovine serum albumin (BSA) (Sigma‐ Aldrich) for 1 h at 37°C. After antigen retrieval by heated citrate buffer, tissues were incubated with primary antibodies mixed with 5% BSA in PBS overnight at 4°C, and subsequently with secondary antibodies for 2 h at 37°C. Slides were washed five times with PBS and incubated with ABC solution. The primary immunohistochemical antibodies included OV6 (#MAB2020, R&D systems), EpCAM (#ab213500, Abcam, Cambridge, England), SOX9(#ab185230, Abcam), SOX2(#ab97959, Abcam), SIRT1 (#ab189494, Abcam), and PCNA (#60097‐1‐Ig, Proteintech).

### 2.5 Immunofluorescent staining

The paraffin‐embedded tissue sections were subjected to deparaffinization and rehydration. Sections were blocked in 5% BSA for 1 h at 37°C. After antigen retrieval by heated citrate buffer, tissues were permeabilized and incubated with primary antibodies. The treated cells were washed twice with PBS and fixed with paraformaldehyde for 15 min. Next, the cells were blocked with 5% BSA for 1 h and then incubated with primary antibodies at 4°C. After an overnight incubation with primary antibodies, the tissue sections or cells were washed with PBS and then labeled with FITC/TRITC‐conjugated secondary antibody for 1 h at 37°C. The cells were further stained with DAPI to visualize the nuclei. The slides and cells were then analyzed under a fluorescent microscope (Nikon Corp, Tokyo, Japan). The primary immunofluorescent antibodies included EpCAM (#ab213500, Abcam), Caspase‐3(#ab179517, Abcam), SOX2(#ab97959, Abcam), and SIRT1 (#ab189494, Abcam). Secondary antibodies were purchased from Proteintech.

### 2.6 Flow cytometry analysis

Cell apoptosis was examined using Annexin V‐FITC Apoptosis Detection Kits (BD Pharmingen). Briefly, the cells treated with different concentrations of capsaicin were harvested by centrifugation(4°C, 1000 rpm, 5 min) after trypsinization. The cell pellet was washed with PBS and resuspended in 1× binding buffer. Next, the cell pellet was stained with Annexin V‐FITC and propidium iodide (PI) according to the manufacturer's protocol. The results were analyzed using FlowJo software.

### 2.7 Cell counting kit‐8 assay

For the CCK8 assay (Dojindo Laboratories), cells were seeded in 96‐well plates at a density of 0.5 × 10^4^ cells/well. After incubation with different doses of capsaicin for the indicated time, the cells were treated with 100 μl of DMEM containing 10 μl of CCK8 for 1 h before measurement, using a microculture plate reader at a wavelength of 450 nm.

### 2.8 Colony formation assay

WB‐F344 cells were cultured in a six‐well plate (5 × 10^2^ cells/well) with PBS (control) or capsaicin. After incubation at 37°C for 7 days, the cells were fixed with paraformaldehyde and stained with 0.1% crystal violet solution.

### 2.9 Sphere formation assay

After accurate cell counting, 5 × 10^3^ WB cells in 25 μl of DMEM/F12 medium (5%FBS, 50 ng/ml epidermal growth factor (Sigma) and 1 μg/ml insulin‐like growth factor(Sigma)) mixed with 25 μl Matrigel(BD Pharmingen) were added to a low attachment 96 wellplate (SorfaBio, Beijing, China). The 96 well plate was placed in the incubator for an hour and then added with 100 μl medium. After 3 days, 200 μl culture medium was replenished and added with different doses of capsaicin. The medium was changed every 2 days. Spheres were observed and captured.

### 2.10 Transient transfection with SIRT1 and SOX2 overexpression plasmid

Transient transfections with plasmid vector (HanBio Technology) were performed using lipofectamine (HanBio Technology) according to the instructions supplied by the manufacturer. The rat SIRT1 or SOX2 gene was cloned into a pcDNA3.1‐EF1a‐mcs‐3flag‐CMV‐GFP plasmid. After 48 h of transfection, the cells were harvested or treated with capsaicin for the purpose of the experiment.

### 2.11 Immunoprecipitation and western blotting

Immunoprecipitation (IP) was performed using whole cell protein lysates obtained from trypsinized cells using the Pierce Classic IP Kit (Thermo Scientific). The lysates were resuspended in RIPA buffer supplemented with protease inhibitors (Thermo Scientific) and/or deacetylase inhibitor (Beyotime), incubated on ice for 30 min, and centrifuged for 20 min at 14 000 ***g***. The proteins were boiled for 5 min and then subjected to SDS‐PAGE before transferring to the PVDF membrane under standard western blotting conditions. The membranes were then blocked in 5% milk/TBST for 1.5 h at room temperature and incubated with primary antibodies overnight at 4°C. It was followed by the incubation with secondary antibodies (1:5000 in blocking buffer) at room temperature for 2 h. Proteins were visualized using ECL (Millipore). The primary antibodies used in this study included BCL2(#60178‐1‐Ig, Proteintech), β‐actin(#20536‐1‐AP, Proteintech), ubiquitin(#10201‐2‐AP, Proteintech), ɑ‐tubulin(#11224‐1‐AP, Proteintech), histone H3 (#17168‐1‐AP, Proteintech), total Caspase‐3(#14220, Cell Signaling Technology), cleaved Caspase‐3 (##9661, Cell Signaling Technology), SOX2(#ab97959, Abcam; #sc‐365,823, Santa Cruz), SIRT1 (#ab189494, Abcam), NANOG(#14295‐1‐AP, Proteintech), OCT4(#ab18976, Abcam), c‐Myc (#ab32072, Abcam), and acetylated lysine (#9441S, Cell Signaling Technology). Secondary antibodies were purchased from Proteintech. For quantification, signals were densitometrically normalized to β‐actin/ɑ‐tubulin/ histone H3 by Image J analysis.

### 2.12 Preparation of cytosolic and nuclear protein extracts

Cytosolic and nuclear fractions of cells were prepared according to the manufacturer's protocols using a nuclear extraction kit (Beyotime). Briefly, WB‐F344 cells were treated with capsaicin for 4 h, washed twice with ice‐cold PBS, and harvested with a hypotonic buffer containing phosphatase inhibitors (Thermo Scientific). After incubation at 4°C for 15 min and centrifugation at 14,000 g for 1 min at 4°C, the supernatants (cytoplasmic fraction) were collected. For the separation of nuclear fractions, cell pellets were resuspended in lysis buffer and incubated at 4°C for 30 min under shaking. After 30 min, the suspension was centrifuged at 14,000 g for 10 min at 4°C and supernatant was collected (nuclear fraction). The cytoplasmic and nuclear fractions were then prepared for western blotting.

### 2.13 Statistical analysis

All experiments were repeated at least three times. Calculations were performed using GraphPad Prism 8.0 (GraphPad Software). Data were presented as mean ± SD. Statistical analyses were performed using Student's *t*‐test and one‐way ANOVA with significance levels of *p < 0.05 and **p < 0.01.

## 3 RESULTS

### 3.1 Capsaicin inhibits DEN‐induced rat liver tumorigenesis

**FIGURE 1:**
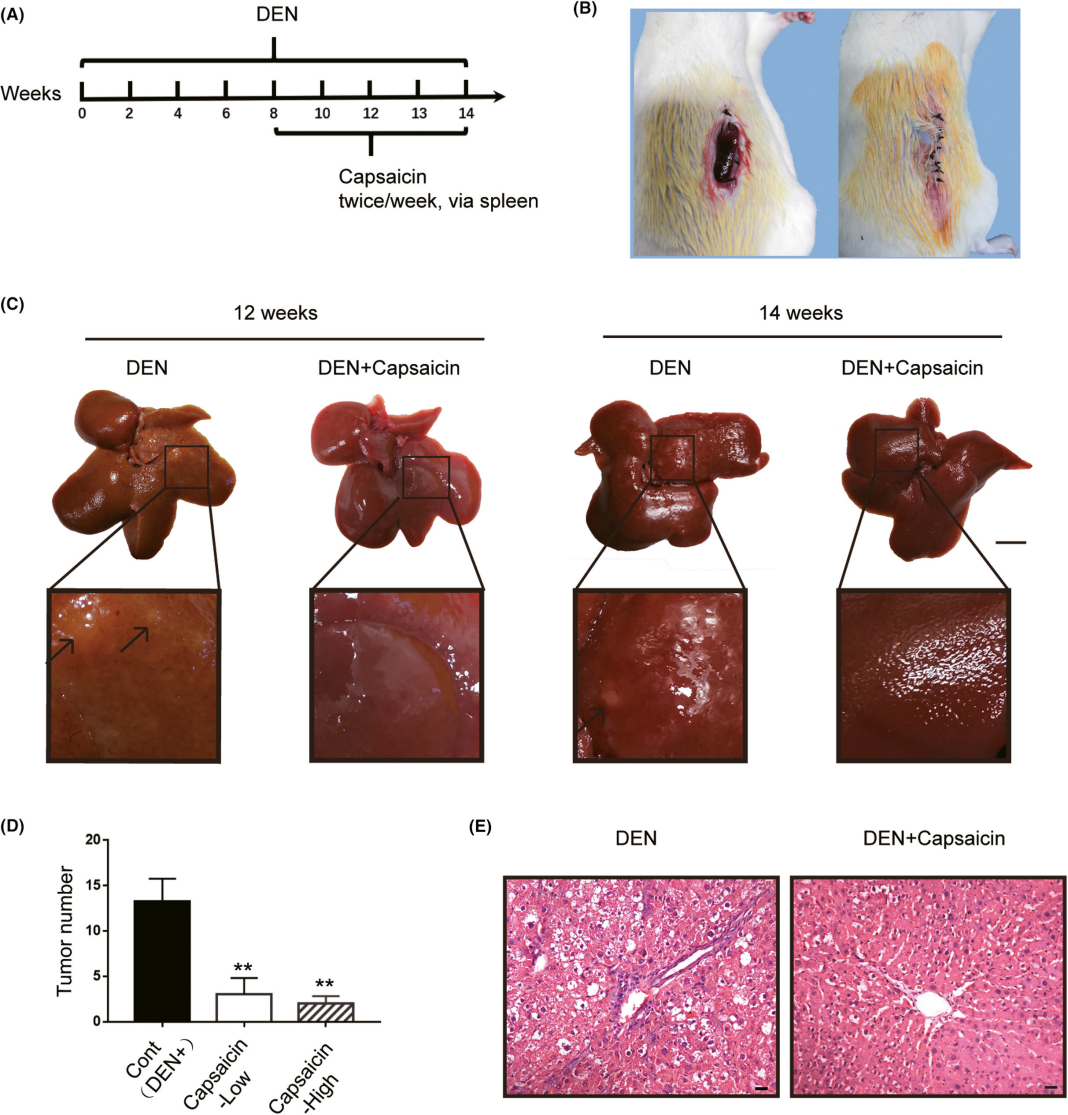
Capsaicin suppressed diethylnitrosamine (DEN)‐induced rat liver tumorigenesis. (A) Schematic diagram of the liver tumor model establishment and capsaicin administration. (B) Subcutaneous transposition of the spleen in rats. (C) The morphology of the liver in different rat groups. Scale bars, 1 cm. (D) The liver of the DEN‐induced rat treated with capsaicin presented a reduced number of surface tumor nodules. (*n* = 4). (E) Hematoxylin and eosin staining in the liver of the DEN‐induced rat model treated with capsaicin showed reduced necrosis, cellular edema, and nuclear atypia. Scale bars, 20 μm (*p < 0.05; **p < 0.01)

Although the anti‐HCC effect of capsaicin has been proven in vitro,^10^, ^20^ its corresponding potential in vivo has rarely been evaluated because of the limitations of delivery methods and their irritation potential. The DEN‐induced rat HCC model constitutes the most widely used experimental model for studying hepatocarcinogenesis. To evaluate the biological effect of capsaicin on hepatocarcinogenesis in vivo, we treated the DEN‐induced rat liver cancer model with capsaicin‐loaded liposomes and established a control group (DEN+) without such treatment (Figure 1A). Furthermore, due to the strong irritation potential of capsaicin, we delivered the liposomal capsaicin through the subcutaneous transposition of the spleen (Figure 1B). Liposomal capsaicin treatment provided significant protection against hepatocarcinogenesis at 12–14 weeks after the DEN treatment (early stage). Representative images of excised livers at 12 weeks are presented in Figure 1C, and the corresponding numbers of counted surface nodules are shown in Figure 1D. Compared to the control group (DEN+), the liver treated with capsaicin showed few tumor nodules and was characterized by a smooth surface. Moreover, hematoxylin and eosin (H&E) staining in the DEN+capsaicin group showed a lower level of liver injury, including swelling, inflammatory cell infiltration, and necrosis (Figure 1E). These observations indicate that capsaicin has a significant impact on liver tumorigenesis in rats.

### 3.2 Capsaicin inhibits HPCs activation and induces its apoptosis during tumorigenesis

**FIGURE 2:**
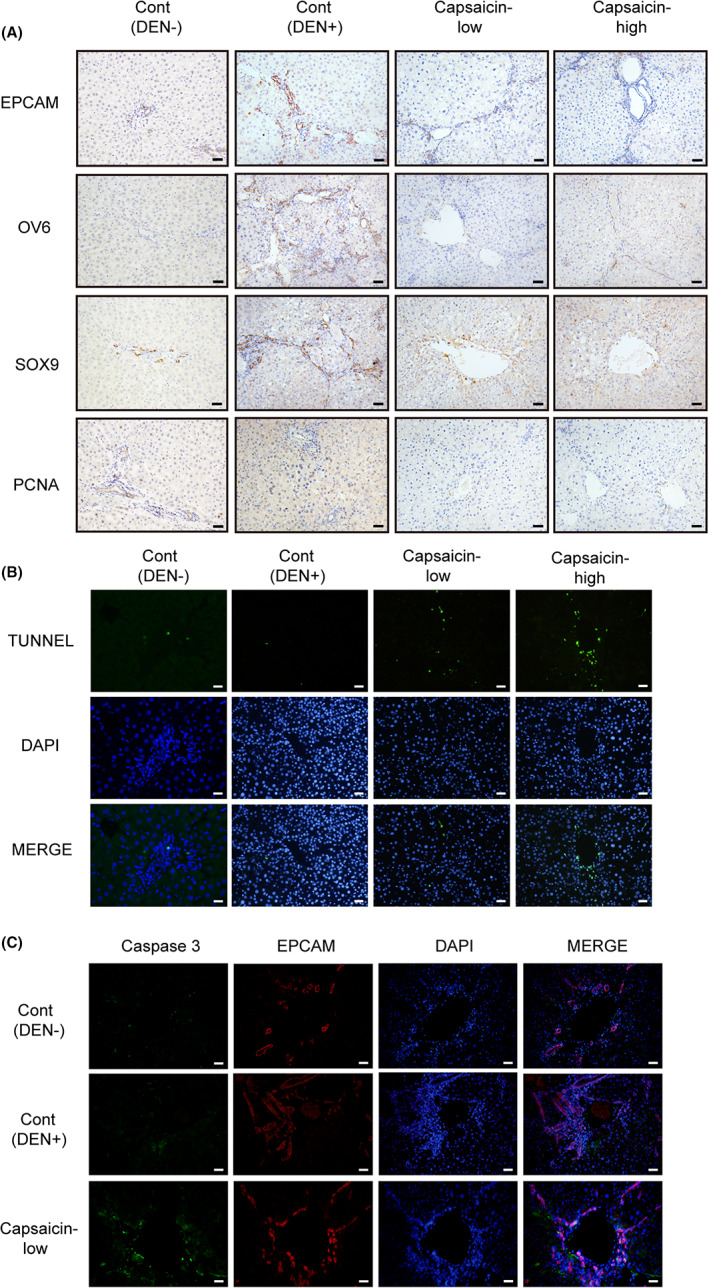
Capsaicin reduced hepatic progenitor cells (HPCs) activation, and induced HPCs apoptosis in the liver of diethylnitrosamine (DEN)‐induced rats suffering from hepatocellular carcinoma. (A) Immunohistochemistry staining showed that the expression of HPCs markers, EpCAM, OV6, SOX9 as well as proliferation marker PCNA was decreased in the DEN+capsaicin group compared to the DEN group. (B) Tunnel staining showed a higher number of apoptotic cells in the DEN+capsaicin group than that in the DEN group. (C) Immunofluorescence co‐staining of Caspase‐3 and EpCAM suggested capsaicin‐induced HPCs apoptosis in DEN‐treated rat liver. Scale bars, 40 μm

To identify how capsaicin decreased DEN‐induced liver tumor generation and how it affected the status of HPCs, we performed immunohistochemistry analysis. Representative images are shown in Figure 2A and Figure S1A. Collectively, we found that the levels of HPCs markers (EpCAM, SOX9, and OV6) as well as the proliferation marker PCNA, were significantly reduced compared to the control (DEN+) group, indicating that capsaicin significantly inhibited HPCs activation. Moreover, the tunnel assay showed apoptosis specifically around the tubular structure, suggesting that capsaicin treatment induced apoptosis in HPCs around bile duct (Figure 2B and Figure S1B). Furthermore, we performed immunofluorescence co‐staining of Caspase‐3 and EpCAM, which validated that capsaicin‐induced apoptosis of HPCs in the DEN‐HCC rat model (Figure 2C and Figure S1C).

### 3.3 Capsaicin induces human HepG2 cell apoptosis but not that of rat WB‐F344 cells

**FIGURE 3:**
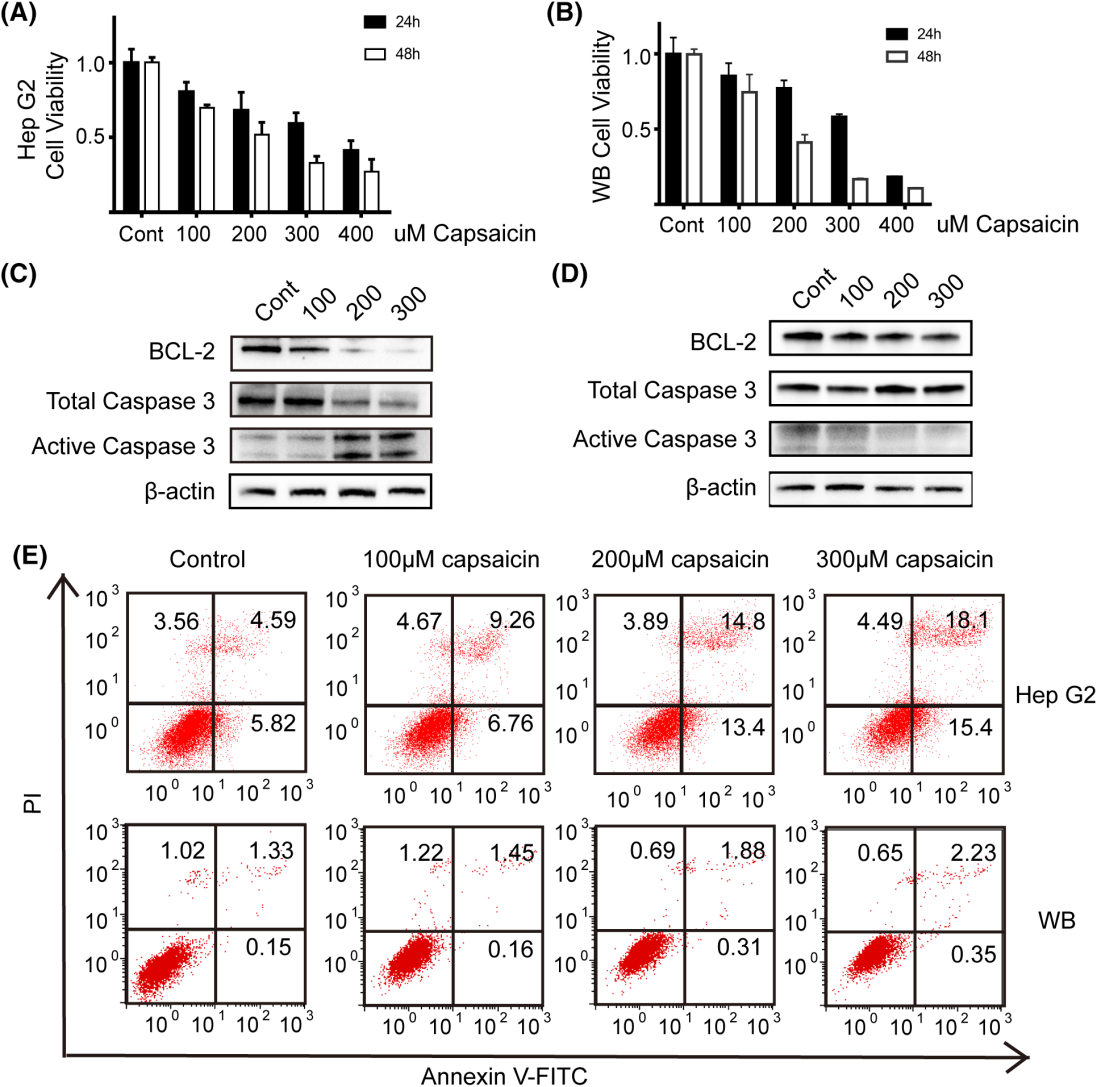
Capsaicin induced apoptosis in HepG2 but not in WB‐F344 cells. (A) and (B) Capsaicin inhibited the proliferation of HepG2 cells (A) and WB‐F344 cells (B) as detected with the CCK‐8 assay (*n* = 5). (C) and (D) The expression of apoptosis‐related proteins BCL2 and Caspase‐3 was detected with western blotting in HepG2 cells (C) and (D) WB‐F344 cells incubated with different doses of capsaicin (0, 100, 200, 300 μmol/L) for 24 h. (E) Flow cytometric analysis validated that capsaicin induces human HepG2 cell apoptosis significantly but not that of rat WB‐F344 cells

Capsaicin induces apoptosis in various cancer cell lines.^6^, ^7^ To further determine whether capsaicin decreased liver tumor incidence by inducing apoptosis, we examined apoptosis in the human liver cancer cell line HepG2 and rat normal hepatic progenitor cell line WB‐F344 in vitro. We treated HepG2 cells (Figure 3A) and WB‐F344 cells (Figure 3B) with increasing doses of capsaicin for 24 and 48 h. Time‐dependent and dose‐dependent inhibition of cell viability in both HepG2 and WB‐F344 cell lines, as measured with the CCK8 assay, is presented in Figure 3A, B (*p* < 0.05). Consistent with a previous study,^20^ capsaicin caused apoptosis in HepG2 cells, which was verified by western blotting (Figure 3C). However, capsaicin had no significant apoptotic effect on WB‐F344 cells (Figure 3D). We validated this finding using flow cytometric analysis (Figure 3E). These results revealed that capsaicin had no apoptotic effect on WB‐F344 cells, but there was a significant decrease in the proliferation of WB‐F344 cells.

### 3.4 Capsaicin inhibits abnormal activation of HPCs related to hepatocarcinogenesis via downregulating SOX2

**FIGURE 4:**
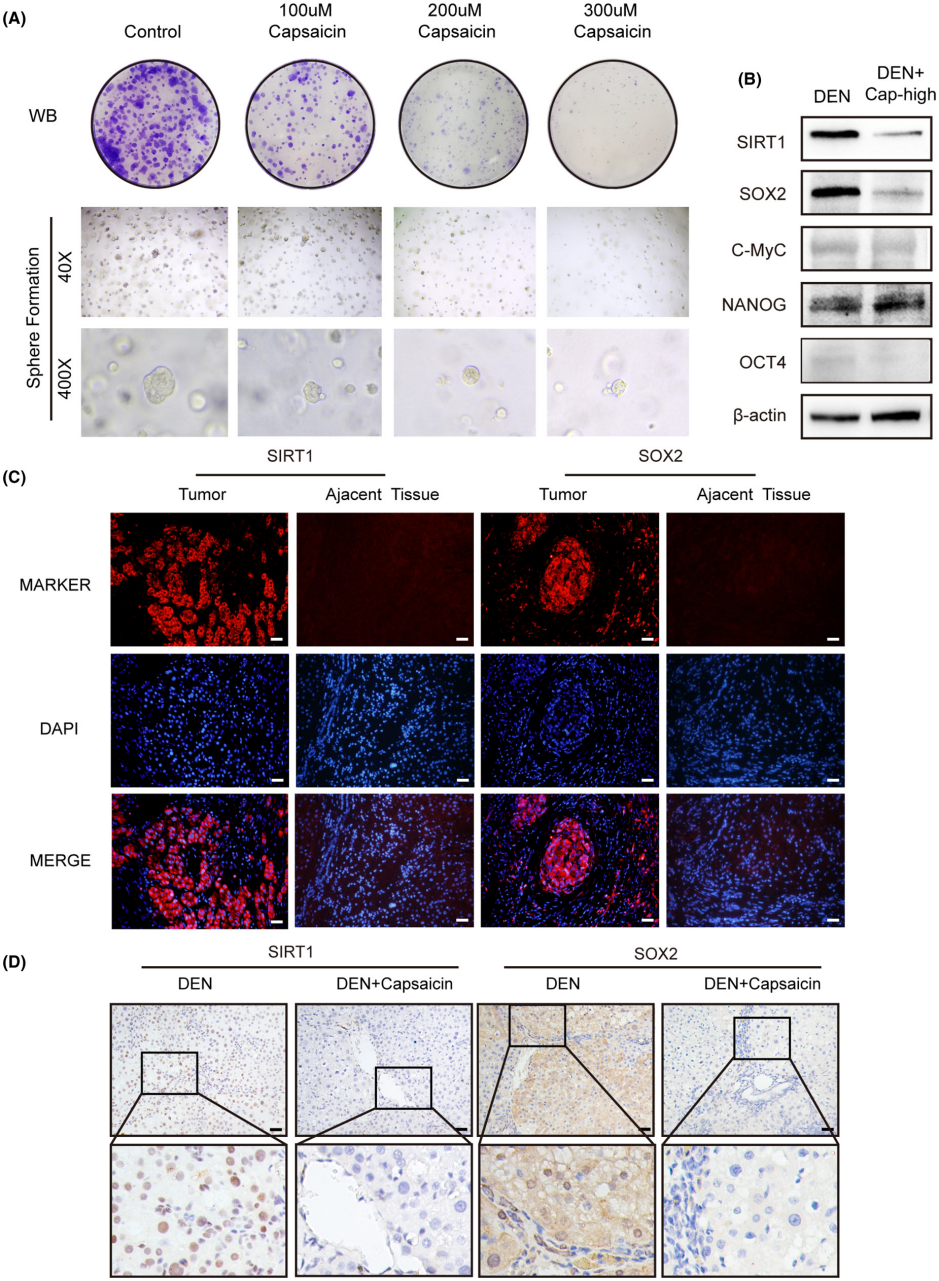
(A) Capsaicin inhibited the colony formation and sphere formation of WB‐F344 cells in a dose‐dependent manner. Colonies were fixed with 4% paraformaldehyde and stained with 0.1% crystal violet 7 days later. Spheres were observed and captured 6 days later. (B) The expression of OCT4, SOX2, c‐Myc, NANOG, and SIRT1 was detected with western blot in liver tissues from the DEN and DEN+capsaicin groups. (C) Immunofluorescence staining showed that SIRT1 and SOX2 were expressed in human hepatocarcinomaat a higher level than in corresponding adjacent tissues. Scale bars, 40 μm. (D) Immunohistochemical staining showed that the expression of SIRT1 and SOX2 was lower in the DEN+capsaicin group than in the DEN group. Scale bars, 40 μm

Research has shown that capsaicin has a differential effect on cancer and noncancer cells.^21^ Interestingly, the study demonstrated that capsaicin induces protective autophagy in noncancerous cells while exerting cytotoxic apoptotic effect in cancer cells. Consistent with this report, our study provides partial support for the idea that capsaicin does not play a lethal role on noncancerous cells. Capsaicin suppressed HPCs activation and induced apoptosis in the DNE‐induced pro‐inflammatory tumor microenvironment in model rats. However, the compound did not exert an apoptotic effect on noncancerous rat cell line WB‐F344 under a routine culture environment. Therefore, we postulated that capsaicin might decrease the stemness of HPCs. As illustrated in Figure 4A and Figure S1D, capsaicin inhibited the stemness of WB‐F344 cells in the clonal formation assay and sphere formation assay. To further determine the specific regulators for maintaining the stem cell properties of HPCs, we examined the levels of several known stem cell protein markers, OCT4, SOX2, NANOG, and c‐Myc in rat liver tissue. Notably, we found that only the SOX2 level significantly decreased after capsaicin treatment in the DEN‐induced rat HCC when compared to the control (DEN+) (Figure 4B). Therefore, the results suggest that SOX2 reduction inhibits abnormal activation of HPCs and impairs hepatocarcinogenesis.

### 3.5 SIRT1 and SOX2 interact to regulate the stemness of HPCs in vivo

Given the importance of SOX2 in maintaining the stemness of HPCs, it is crucial to understand the mechanism of capsaicin on regulating SOX2 expression and activity in HPCs. Both transcriptional and post‐translational regulation of SOX2, including phosphorylation, ubiquitination, acetylation, SUMOylation, and methylation, have been extensively studied.^11^, ^13^, ^22^ However, it remains unclear whether changes in post‐translational modifications impair SOX2 stability during capsaicin treatment in HPCs. SIRT1 is a protein which promotes hepatocarcinogenesis. It could regulate SOX2 by deacetylation to maintain stemness during mouse somatic reprogramming.^15^, ^17^, ^18^ Therefore, SIRT1 may be a strong candidate for maintaining SOX2 stability via post‐translational modification in HPCs.

To determine whether SIRT1 and SOX2 interact to regulate hepatocarcinogenesis, we performed immunofluorescence staining. As shown in Figure 4C, our findings indicated that SIRT1 and SOX2 were upregulated in human liver cancer tissue sections compared to the adjacent tissue. This implies that SIRT1 and SOX2 are involved in the signaling pathway underlying hepatocarcinogenesis. Meanwhile, immunohistochemistry analyses for SIRT1 and SOX2 revealed less intense staining of capsaicin‐treated rat HCC liver when compared with the untreated control (Figure 4D), which was additionally confirmed by western blot results (Figure 4B). These findings suggest that the regulation of SOX2 by SIRT1 is likely to be the mechanism involved in the effect of the capsaicin treatment in HPCs.

### 3.6 SIRT1 and SOX2 interact to regulate the stemness of HPCs in vitro

**FIGURE 5:**
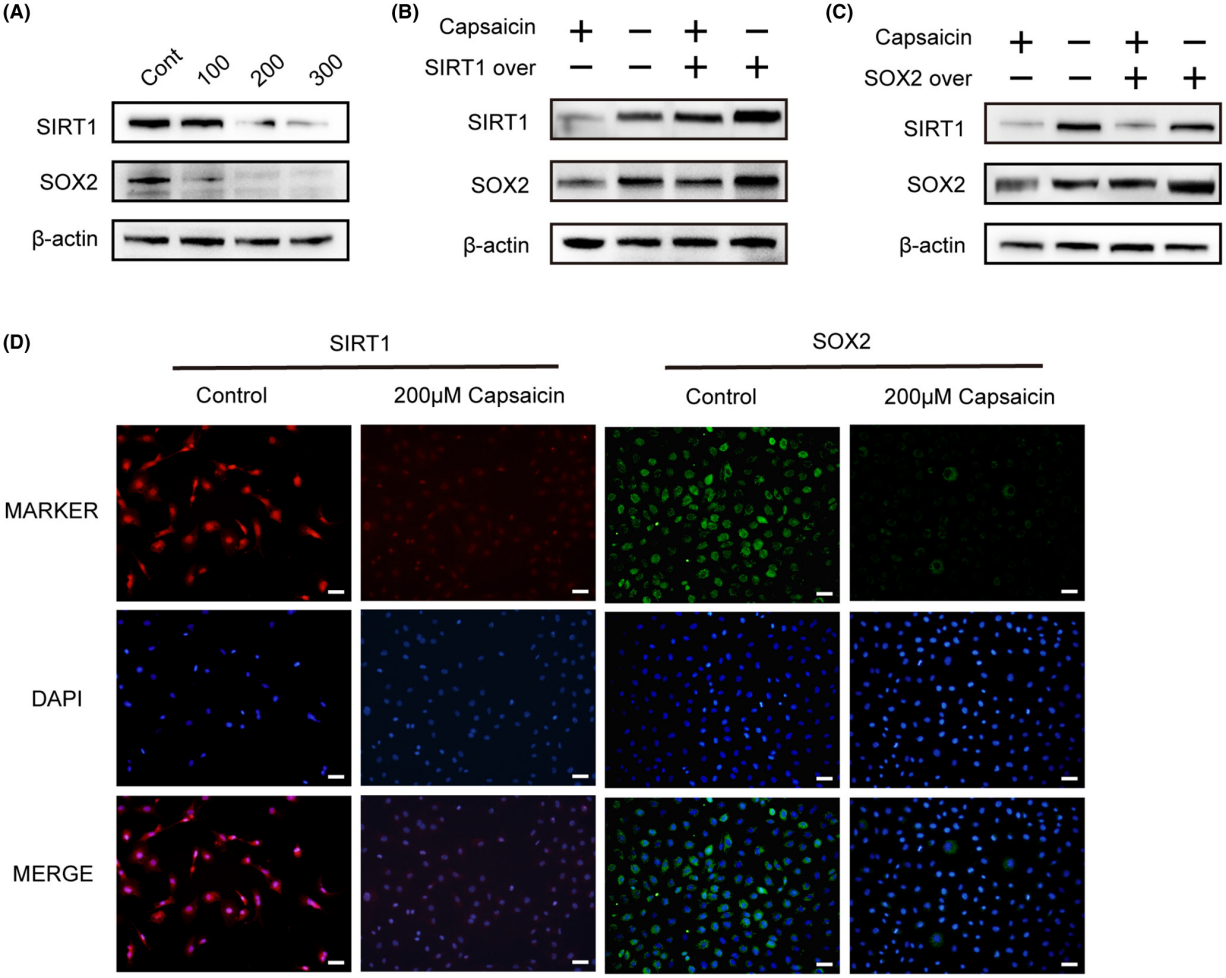
(A) Capsaicin caused a dose‐dependent reduction in SIRT1 and SOX2 levels as detected with western blotting. WB‐F344 cells were treated with different concentrations (0, 100, 200, and 300 μM) of capsaicin for 24 h. β‐actin served as a loading control (*n* = 3). (B) Overexpression of SIRT1 induced by plasmid transfection can compensate for the reduction of SOX2 levels caused by capsaicin treatment in WB‐F344 cells. (C) Overexpression of SOX2 induced by plasmid transfection cannot compensate for the SIRT1 reduction induced by capsaicin treatment in WB‐F344 cells. (D) Immunofluorescent staining showed that the expression of SIRT1 and SOX2 was decreased by capsaicin treatment. Scale bars, 40 μm

To investigate the role of capsaicin in regulating SIRT1 and SOX2, we first estimated SIRT1 and SOX2 protein expression in vitro. Consistent with previous in vivo results (Figure 4), western blot analysis revealed that WB‐F344 cells exposed in vitro to the increasing dose of capsaicin (100–300 μM) for 24 h, exhibited downregulated expression of both SOX2 and SIRT1 in a dose‐dependent manner (Figure 5A). To gain insights into the possible SIRT1/SOX2 signaling pathway, we performed plasmid transfection to overexpress SIRT1 and SOX2 in WB‐F344 cells. As shown in Figure 5B–C, overexpression of SIRT1 remarkably increased the expression of SOX2 protein, while overexpression of SOX2 alone did not enhance SIRT1 expression. Additionally, SIRT1 overexpression increased the level of SOX2 following capsaicin treatment, compared to cells normally expressing SIRT1. However, overexpression of SOX2 failed to compensate for SIRT1 deficiency. Moreover, the analysis by immunofluorescence staining indicated that 200 μM capsaicin caused a reduction in SIRT1 and SOX2 expression in WB‐F344 cells (Figure 5D).

Altogether, these findings suggest that the SIRT1/SOX2 signaling pathway is involved in the capsaicin‐mediated HPCs inhibition.

### 3.7 Capsaicin inhibits SIRT1, and subsequently acetylated SOX2 is translocated from the nucleus to the cytosol and degraded by ubiquitination

**FIGURE 6:**
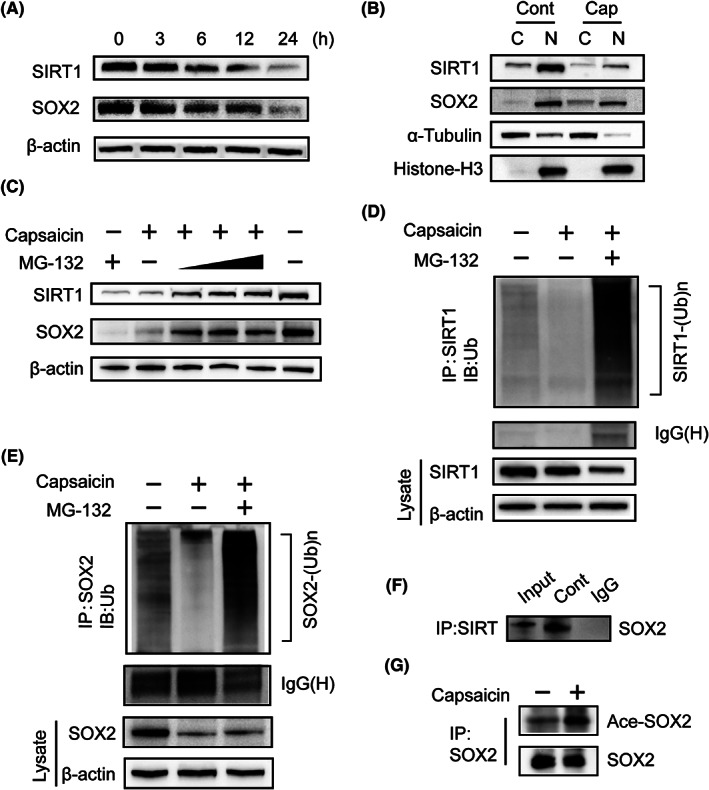
(A) Capsaicin treatment caused a time‐dependent reduction in SIRT1 and SOX2 levels in WB‐F344 cells as detected with western blotting. (B) Following exposure of WB‐F344 cells to capsaicin (200 μM) for 4 h, cytosolic and nuclear extracts were prepared and subjected to western blotting. α‐Tubulin and Histone‐H3 were used as cytosolic and nuclear markers, respectively. (C) WB‐F344 cells were exposed to 200 μmol/L capsaicin alone or in combination with MG‐132 (5 μmol/L, 10 μmol/L, 20 μmol/L) for 3 h, and the protein level of SIRT1 and SOX2 was examined by western blot analysis. (D) and (E) Following treatment of WB‐F344 cells with capsaicin (200 μM) alone or in combination with MG‐132 (20 μM) for 3 h, the ubiquitination of endogenous SIRT1 (D) and SOX2 (E) was determined by immunoprecipitation. (F) SIRT1 was immunoprecipitated from WB‐F344 cells, and western blot analysis was performed using antibodies for SOX2 to investigate direct binding between SIRT1 and SOX2. (G) Following treatment of WB‐F344 cells with capsaicin (200 μM) for 3 h, western blotting detected immunoprecipitated SOX2 with the use of SOX2 antibody and acetylated SOX2 with the use of acetylated lysine antibody to confirm the acetylation status of SOX2

To examine how capsaicin affects SIRT1 protein expression, we exposed WB‐F344 cells to 200 μM capsaicin for different periods of time. Western blot analysis results indicated time‐dependent inhibition of the protein expression of both SIRT1 and SOX2 (Figure 6A). Moreover, the cytosolic and nuclear protein assays revealed that both SIRT1 and SOX2 were abundantly located in the nucleus, but capsaicin significantly reduced their nuclear accumulation compared to the control (Figure 6B). The acetylation of SOX2 induces its nuclear export and subsequent degradation by ubiquitination, decreasing its levels in embryonic stem cells.^22^ Altogether, we hypothesized that capsaicin decreases the expression of SIRT1in HPCs, and subsequently acetylated SOX2 is translocated from the nucleus to the cytosol, and degraded by ubiquitination.

**FIGURE 7:**
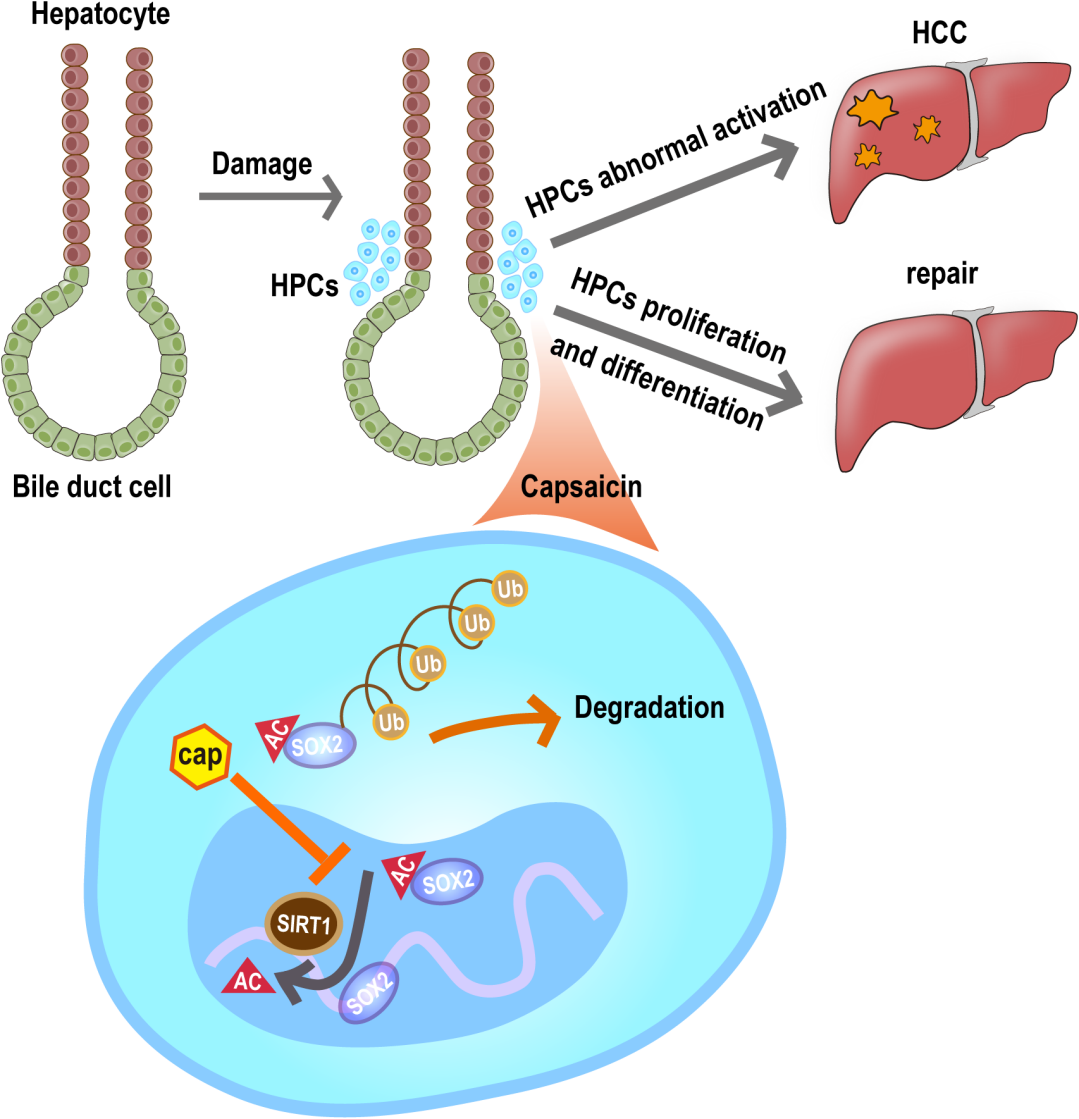
Proposed schematic diagram of capsaicin‐mediated suppression of hepatocellular carcinoma. Hepatic progenitor cells (HPCs) abundantly express both SIRT1 and SOX2 in their nuclei. SOX2 is an important regulator in maintaining the stemness of HPCs and is regulated via deacetylation by SIRT1. After capsaicin treatment, SIRT1 undergoes ubiquitin‐mediated degradation, and subsequently acetylated SOX2 is then transported from the nucleus to the cytosol, where it is also degraded by ubiquitination

To test our hypothesis, we investigated the ubiquitination of SIRT1 and SOX2 in capsaicin‐treated WB‐F344 cells. The western blot results revealed that the inhibitory effect of capsaicin on SIRT1 and SOX2 protein expression was blunted by the proteasome inhibitor MG‐132 (Figure 6C, D–E). This indicates that the capsaicin‐induced downregulation of SIRT1 and SOX2 requires ubiquitin‐mediated degradation. To identify how SIRT1 regulates SOX2, we first performed a co‐immunoprecipitation assay on WB‐F344 cells. We found that SIRT1 regulates SOX2 by direct binding (Figure 6F). To further identify whether the downregulation of SIRT1 after capsaicin treatment increases the acetylation level of SOX2, we performed a co‐immunoprecipitation assay with the SOX2 antibody, followed by western blot analysis using the acetyl‐lysine antibody to detect acetylated SOX2. We found that capsaicin upregulated the acetylation level of SOX2 (Figure 6G). These results indicate that SIRT1 interacts directly with SOX2 and keeps it in the deacetylated state in the nucleus. In conclusion, capsaicin caused a significant reduction in SIRT1, and a subsequent increase in the acetylation level of SOX2, leading to nuclear export and ubiquitination‐dependent degradation of SOX2 (Figure 7).

## 4 DISCUSSION

Capsaicin, the bioactive compound of hot chili peppers, exhibits anticancer, anti‐oxidant, and anti‐inflammatory properties.^10^, ^23^ Growing evidence from in vitro studies indicates that capsaicin acts against several cancers, including HCC.^20^ However, the in vivo mechanism of action of capsaicin in the context of HCC remains unknown. We first investigated the biological function of capsaicin in a DEN‐induced rat model of liver tumorigenesis. In this study, we observed that capsaicin exhibited anticarcinogenic properties in the early stage after 12 weeks of the DEN treatment. Although immunohistochemical staining showed remarkably reduced activation and induced apoptosis of HPCs during DEN intervention in vivo, capsaicin had no significant apoptotic effect on WB‐F344 cells in vitro. Capsaicin possesses two modes of action. It protects normal tissues and acts against cancer cells.^21^ Our results showed that the capsaicin administration resulted in HPCs apoptosis under a DEN‐induced pro‐inflammatory tumor microenvironment in the HCC rat model and the lack of apoptotic effect in a normal rat cell line (WB‐F344) under routine culture environment. Thus, we concluded that capsaicin suppresses tumorigenesis partially by inhibiting the stemness of HPCs. However, there are no previous reports of such a conclusion. This necessitated the identification of potential transcription factors that inhibit the stemness of HPCs in tumor suppression following capsaicin treatment.

SOX2, a pluripotency transcription factor, plays an important role in stemness, and its overexpression is associated with a poor prognosis of HCC patients.^13^, ^24^ SOX2 regulates the renewal of progenitor cells in the human breast and cornea.^25^, ^26^ Notably, in this study, SOX2 was the only transcription factor whose expression levels were significantly altered after capsaicin treatment, unlike NANOG, c‐Myc, and OCT4. These expression profiles were detected both in vivo and in vitro. Accordingly, SOX2 enhances self‐renewal and tumorigenicity potential in liver CSCs.^13^ On the other hand, liver CSCs, probably generated from HPCs, already have the potential for tumorigenicity, self‐renewal, lineage differentiation, and disease recurrence.^27^ These findings suggest that SOX2 plays a role in controlling the abnormal activation of HPCs to regulate HCC generation. We further observed that capsaicin reduced the stemness of the WB‐F344 cell line, as validated by colony and sphere formation assay, and decreased the expression of stem cell transcription factor SOX2.

Although we proposed a possible mechanism for the stemness inhibition of the HPCs, the degradation mechanism of SOX2 remains unclear. Some studies reported that the deacetylation level of SOX2 is regulated by SIRT1 and enhances somatic reprogramming in mice, indicating that post‐translational modifications of SOX2 are important for regulating stemness.^16^ SIRT1 is abundant in liver CSCs and mediates carcinogenesis, tumor invasion, and tumor progression via deacetylation.^28^ Furthermore, SOX2 is the primary downstream regulator of SIRT1‐mediated tumorigenicity in liver CSCs.^13^ Previous studies demonstrated that SIRT1 functions as a promotor of liver cancer and its level is significantly decreased by capsaicin in bladder cancer cells.^29^, ^30^ These observations are important because they suggest a potential mechanism by which SOX2 can be regulated by SIRT1 via post‐translational modification. We demonstrated that SIRT1 and SOX2, colocalized mainly in the nucleus, were highly expressed in both nontreated rats and human HCC tissues, and the protein levels of both decreased after capsaicin treatment in WB‐F344 rat cells. Additionally, an IP assay indicated the regulation of SOX2 by direct interaction with SIRT1. Next, we focused on the degradation of SOX2 via acetylation, nuclear export, and ubiquitination. We found that SOX2 was deacetylated by SIRT1 in the nucleus of WB‐F344 cells. Subsequently, SIRT1 decreased in capsaicin‐treated HPCs. After capsaicin treatment, acetylated SOX2 was translocated from the nucleus to the cytosol, which was followed by proteasomal degradation.

Most natural anticarcinogenic products act by inducing apoptosis, senescence, and oxidative stress in cancer cells. Recent evidences indicate that capsaicin shows anticancer properties against some types of carcinomas acting through apoptosis.^30^, ^31^, ^32^ Although the experimental evidence supports the anti‐hepatocarcinogenic potential of capsaicin, it requires validation in animal models. However, capsaicin possesses strong irritation potential and poor solubility, which limit its mode of delivery as a therapeutic agent. Therefore, we designed a nano‐liposome‐capsaicin molecule for effective delivery into the rat liver. As a result of the subcutaneous delivery of the nano‐liposome‐capsaicin, the local concentration of the drug in the liver increased significantly, the side effects were reduced, and better curative efficacy was achieved. To the best of our knowledge, this is the first study that has been conducted on the anti‐carcinogenic effects of nano‐liposomal‐capsaicin against HCC in a rat model. Consistent with previous findings, capsaicin significantly inhibited hepatocarcinogenesis in a DEN‐induced liver cancer rat model. Moreover, we reported similar results in vitro.

In conclusion, the findings indicate that capsaicin prevents hepatocarcinogenesis by inhibiting the stemness of HPCs. SIRT1 and SOX2 were highly expressed in both rat and human hepatocarcinoma cell lines, and the levels of both proteins significantly decreased following the capsaicin treatment. Subsequently, ubiquitin‐dependent degradation of SIRT1 facilitated the acetylation, nuclear export, and ubiquitin‐dependent proteasomal degradation of SOX2 in WB‐F344 cells. This is the first report to establish a connection between the status of SOX2 acetylation, stemness of HPCs, and hepatocarcinogenesis. This post‐translational modification of SOX2 by SIRT1 provides a potential specific target for the development of HCC therapy.

## CONFLICT OF INTEREST

The authors declare no conflict of interest.

## AUTHOR CONTRIBUTION

In this study, Zhi‐Qin Xie and Hong‐Xia Li performed the experiments and drafted the manuscript. Xiao‐Juan Hou, Ze‐Min Zhu, and Mei‐Yuan Huang helped perform the experiments. Cai‐Xi Tang designed the study, participated in its design and coordination, and helped draft the manuscript. Li‐Xin Wei designed the study and helped perform the experiments. The final manuscript has been reviewed and approved by all the authors.

## ETHICAL APPROVAL STATEMENT

All experiments procedures were conducted according to the protocols approved by the Ethics Committee of Zhuzhou Hospital Affiliated to the Xiangya School of Medicine and Animal Ethics Committee of the Second Xiangya Hospital, Central South University. Informed consent was obtained from all patients.

## Supporting information


Figure S1

## Data Availability

Data Availability Statement All the data generated and/or analyzed during the current study are available from the corresponding author on reasonable request.
