# Supplementary figures and images for: Capsaicin suppresses hepatocarcinogenesis by inhibiting the stemness of hepatic progenitor cells via SIRT1/SOX2 signaling pathway
Source: Cancer Med. 2022 Jun 8;11(22):4283–96. doi: 10.1002/cam4.4777 (PMC9678096; doi:10.1002/cam4.4777)

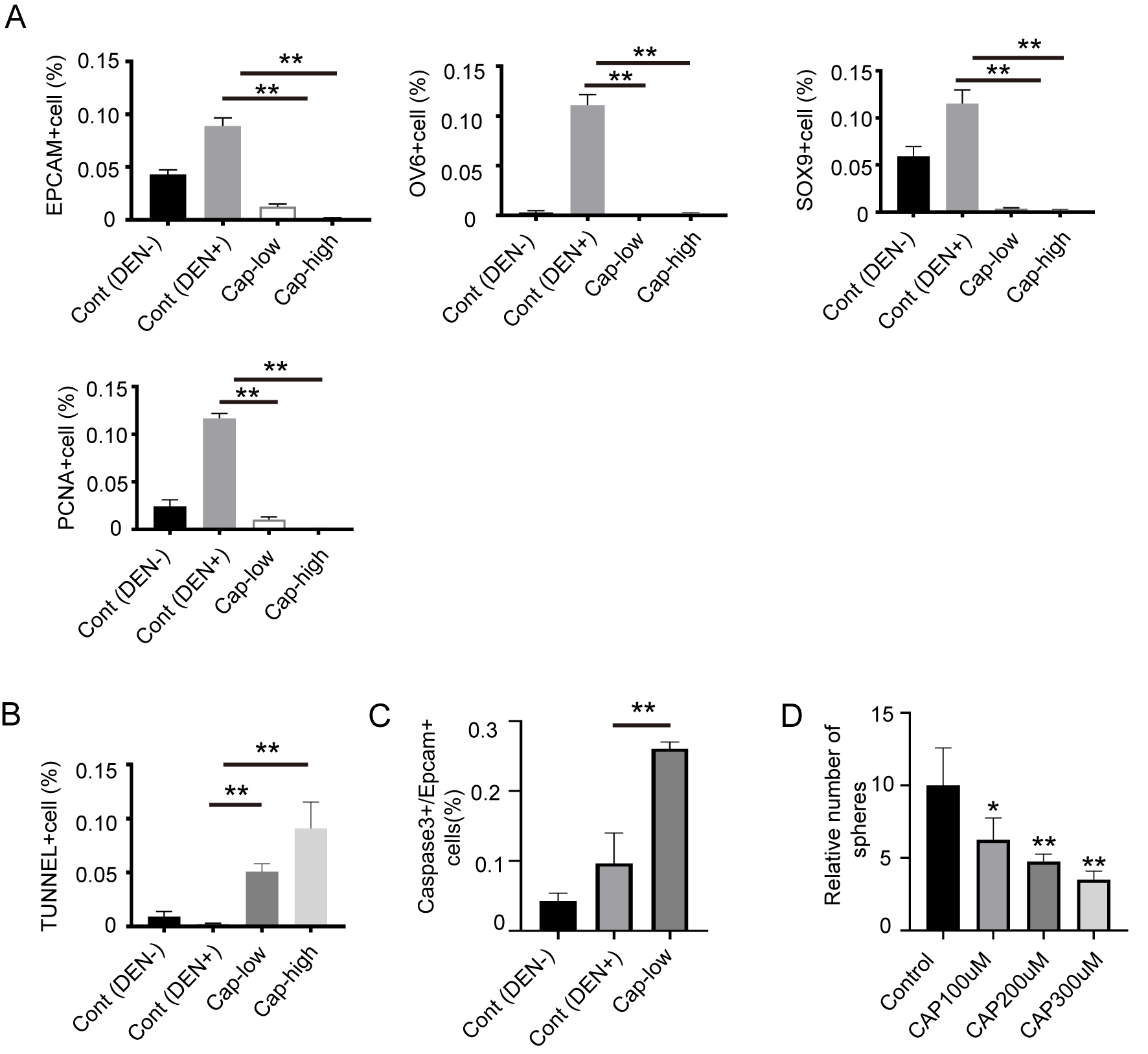

Supplement: Supplementary file 1 — Figure S1 [file CAM4-11-4283-s001.tif]
